# Site-specific dataset of mining and metallurgical residues for resource management

**DOI:** 10.1016/j.dib.2024.110348

**Published:** 2024-03-19

**Authors:** Carlo Cormio, Marta Alonso, Peter Cleall, Soraya Heuss-Assbichler, Daniela Guglietta, Danielle Sinnett, Katalin Szabo, Gorazd Žibret, Teresa Carvalho, Ulrich Kral, Tim Werner, Bruno Lemiere

**Affiliations:** a SERENGEO Srl, *Via* N. Sauro 22, Bologna, BO 40121, Italy; b ICAMCyL Foundation, International Center for Advanced Materials and raw materials of Castilla y León, León Technology Park, Main Building, C/ Julia Morros s/n, First Floor, Offices 106-108, Armunia, León 24009, Spain; c Geoenvironmental Research Centre, School of Engineering, Cardiff University, Cardiff CF24 3AA, UK; d Department of Earth and Environmental Sciences, Ludwig-Maximilians-Universität München, Luisenstraße 37, 80333 Munich, Federal Republic of Germany; e Institute of Environmental Geology and Geoengineering, National Research Council of Italy (CNR IGAG), Research Area of Rome 1, Rome 00010, Italy; f Centre for Sustainable Planning and Environments, University of the West of England, Bristol BS16 1QY, UK; g Mining and Geological Survey of Hungary, Columbus u. 17-23., Budapest H-1145, Hungary; h Geological Survey of Slovenia, Dimičeva ulica 14, Ljubljana SI - 1000, Slovenia; i CERENA, Instituto Superior Técnico, Universidade de Lisboa, Av. Rovisco Pais, Lisboa 1049-001, Portugal; j Technische Universität Wien, Karlsplatz 13, Vienna 1040, Austria; k School of Geography, Earth and Atmospheric Sciences, Faculty of Science, The University of Melbourne, 221 Bouverie Street, Carlton, Victoria, Australia; l BRGM, Orléans F 45060, France

**Keywords:** Resources, Reserves, Mining waste, Secondary raw materials, Tailings, Geodatabase, Circular economy, Resource assessment

## Abstract

This geospatial dataset provides a compilation of findings from an evidence-based review of site-specific resource assessments of mining and metallurgical residues. Information pertaining to location, target material, geological knowledge, extractability, resource classification and stakeholder perspectives was collected from publicly available reports, articles, academic theses, and databases. The dataset

includes 44 relevant data attributes from 64 mining and metallurgical sites in 27 countries. Resource classification is available for 38 sites. The dataset can be used by evaluators of recovery projects, authorities that provide permits, as well as by decision makers in support of developing regulatory policies. The dataset facilitates future addition of sites by the research community and can be further used as a starting point to bridge the estimates on recoverable quantities to the United Nations Framework Classification (UNFC). The UNFC is a universally applicable scheme for the sustainable management of all energy, primary and secondary mineral resources. Its use is stimulated by the European Commission and is intended to be adopted by geological surveys to harmonize the data on the availability of primary and secondary raw materials in Europe in future.

Specifications Table

**Table:** 

Subject	Economic Geology
Specific subject area	Assessment of raw material recoverability from mining and metallurgical residues.
Type of data	Geodatabase Raw, Processed
Data collection	Data were acquired by reviewing publicly accessible information sources for selected sites of mining and metallurgical residues. First, 44 distinctive parameters were defined and grouped into 3 categories and 10 sub-categories. Second, information for each parameter was collected for each of the 64 sites and online spreadsheets were used to compile the data. Third, geographical coordinates were attributed to each storage site and verified by visual identification in satellite imagery.
Data source location	The location of the resource assessment studies, which have been used to produce the dataset, are presented in the geopackage layer “bibliography” [1]. Resource assessment studies are publicly accessible and include the following formats: • Technical reports for investors, based on the Committee for Mineral Reserves International Reporting Standards (CRIRSCO) and United Nations Framework Classification for Resources (UNFC), respectively; • Reports, conference presentations and databases of research projects dealing with production of raw materials from mining and /metallurgical residues; • Reports from international organizations and research centers; • Scientific papers published in international journals; • Doctoral and Master theses; • Online databases on mining sites;
Data accessibility	Repository name: Zenodo Data identification number: 10.5281/zenodo.10029403 Direct URL to data: https://doi.org/10.5281/zenodo.10029403 Instructions for accessing these data: The dataset can be accessed *via* Zenodo data repository.

## 1 Value of the Data

• The geospatial dataset provides a systematic overview of 64 actual estimates of recovery potential from mining and metallurgical residues around the globe. The dataset is valuable as it offers a first perspective on the current practice of resource assessments, which are used to develop recovery projects from the early stage of prospection to the final stage of production.
• The compilation of site-specific information can benefit experts involved in the preparation of estimates of recovery potential, businesses involved in the management of recovery project portfolios and decision-makers involved in the development of regulatory policies for mining, waste management and national resource accounting.
• The database includes 38 site-specific resource estimates based on the Committee for Mineral Reserves International Reporting Standards (CRIRSCO). These resource estimates, in combination with the UNFC Bridging Document, can be further used to classify the recovery projects based on the UNFC.

## 2 Background

The mining sector uses resource assessments to estimate the availability of raw materials from a site. Mine sites, situated on enriched geological occurrences in the earth's crust, host various extractive and ore processing residues in tailings, stockpiles and waste rocks. Such residues, often regarded as wastes, may contain resources available for future extraction. The results of individual resource assessments are difficult to compare because different types of resource classifications and reporting standards have evolved over time to meet sectoral and national needs. Additionally, environmental-socio-economic considerations are needed to assess extractability, but such factors often require assumptions on future impact that can vary between sites. These issues can hamper the creation of recovery project inventories that are needed for national and global resource management. Against this background, this geospatial dataset compiles the findings from an evidence-based review of 64 site-specific resource assessments of mining and metallurgical residues. The harmonized data structure enables the analysis of commonalities and differences among resource assessments to pave the way towards comparative estimates on the future availability of raw materials.

## 3 Data Description

### 3.1 Geopackage Structure

The data repository includes the Mining and Metallurgical Residue Database “mmrdb.qpkg” in GeoPackage format [1]. The coordinate reference system (CRS) is WGS 84, EPSG code: 4326 (https://epsg.org/home.html). The “mmrdb.qpkg” contains 7 layers (see Table 1).

**Table 1:** Layers of the GeoPackage file “mmrdb.qpkg”.

Layer name	Description	Layer type
sites	The layer includes 64 storage sites and an attribute table with 44 attribute fields.	Geometry data [point features]
sites_specs	The layer includes the definitions of the 44 attribute fields of the “sites” layer.	Delimited text file
sites_specs_info	This layer defines the attribute fields of the “sites_spec” layer. It is also shown in Table 3.	Delimited text file
bibliography	The layer includes bibliographic data of the literature used to compile the attribute data of the “sites” layer.	Delimited text file
bibliography_info	This layer defines the attribute fields of the “bibliography” layer. It is also shown in Table 4.	Delimited text file
sitesbiblink	The layer allows the linkage of layers “site” and “bibliography” by primary keys “IDSite” and “IDLiterature”.	Delimited text file
continents	The layer covers the administrative boundaries at the country level of the world. The original layer was retrieved online [2] and was modified adding an attribute column for continents. It was used to produce Fig. 1.	Geometry data [Polygon (Multi-Polygon) features]

Notes: “-“ = not relevant, “GPKG” = Geopackage file format.

**Fig. 1:**
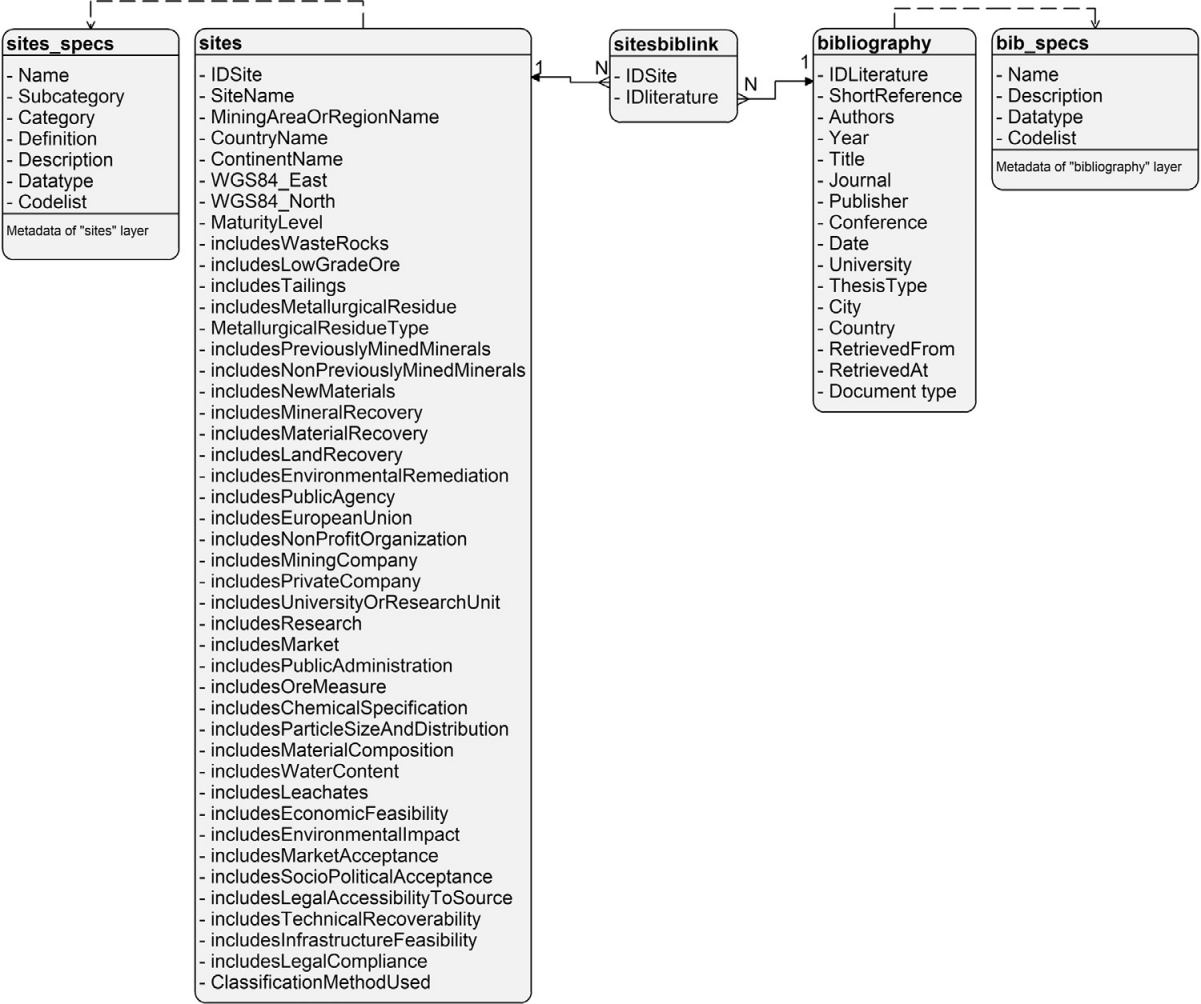
Unified Modeling Language (UML) diagram for the GeoPackage file “mmrdb.qpkg”.

The Unified Modeling Language (UML) diagram (Fig. 1) documents the database structure and content. The UML diagram “UML_diagram.jpg” is also included in the data repository at Zenodo.

### 3.2 Geopackage Layer “Sites”

#### 3.2.1 General Description

Mining and metallurgical residues are the largest waste streams globally. The residues come from the extraction and processing of mineral resources as well as from metallurgical processing. Due to their costly handling, management and storage, they are classified as overburden, waste rock, mine spoils and tailings. Slags and ashes are specific categories at sites with metallurgical production facilities. The classification is based on grain size, on residual economic value (waste rock is an ore below economic grade at the time of mining), and potential risk after storage. Overburden is used for different engineering applications such as backfilling, waste facility coverage and slope stabilisation. Waste rock stockpiles and heaps can be installed temporarily or permanently. Permanent installations are only acceptable if they do not cause human and environmental harm. Tailings are stored in ponds surrounded by dams or are used for mine backfill after potential treatment (e.g. filter pressing) beforehand. Slags and ashes are often stacked in heaps or (sanitary) landfills.

Waste rocks, tailings and slags still contain lower grades of the mined commodity, which may become valuable as economic ore grades steadily decrease. Such residues may be subsequently re-mined, as material extraction is cheaper and less energy-consuming compared to primary ores from open pits, underground and deep-sea mining. They potentially contain various other commodities of economic interest (e.g. precious or critical raw materials), which have not been targeted at the time of initial mining. This is especially valid for historic mine waste sites.

**Fig. 2:**
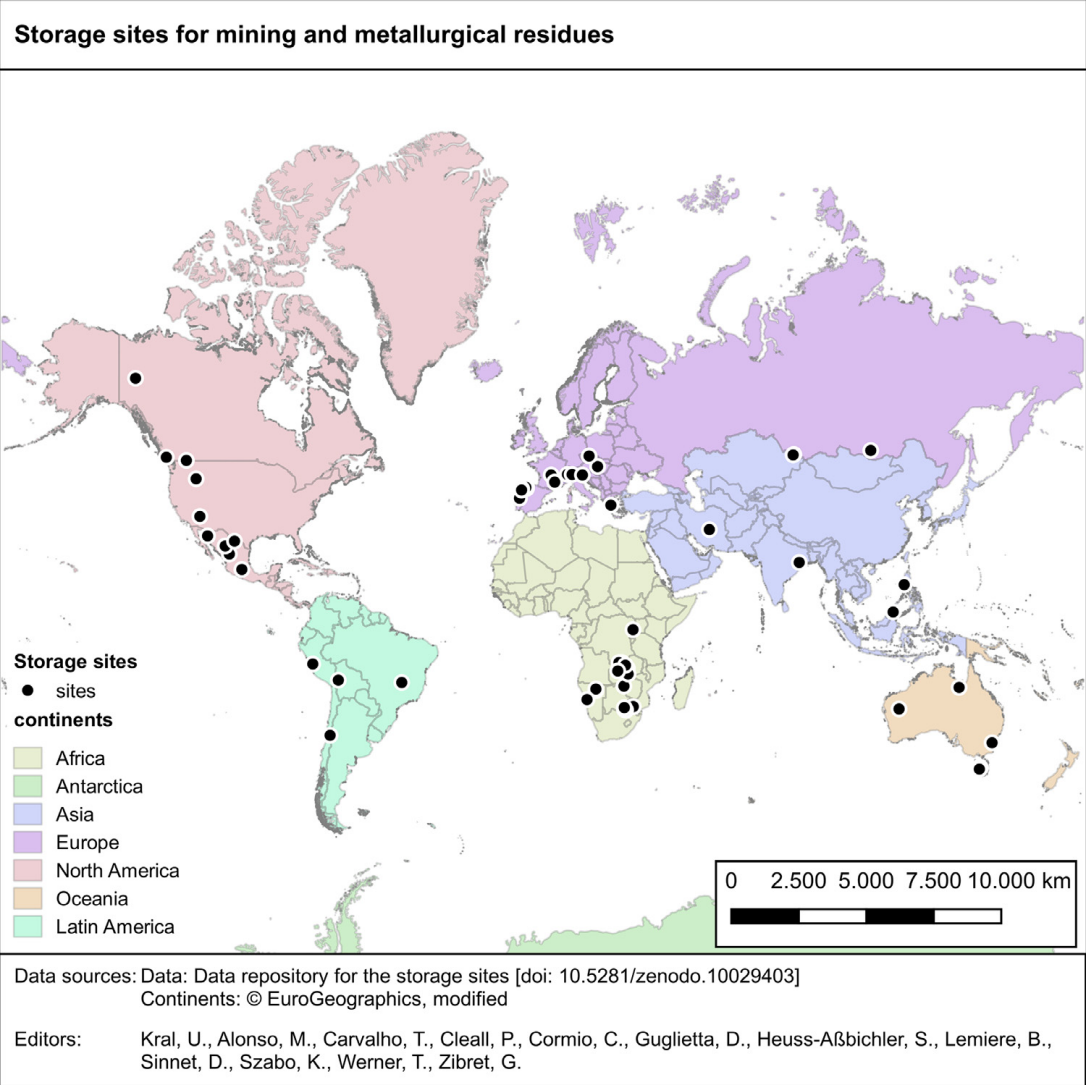
Geographical location of mining and metallurgical sites that are included in the geospatial dataset (sites layer), mapped by continent.

The “sites” layer includes information on 64 residue sites [1]. These sites have been selected to demonstrate the current practice of resource recovery assessments without a claim to capture all storage sites across the globe. However, the grouping of the 64 storage sites by continent shows a global distribution with 28% of sites in Africa, 27% in Europe, 16% in North America, 9% in Oceania, 11% in Asia and 9% in Latin America (Fig. 2).

#### 3.2.2 Attribute Table

**Fig. 3:**
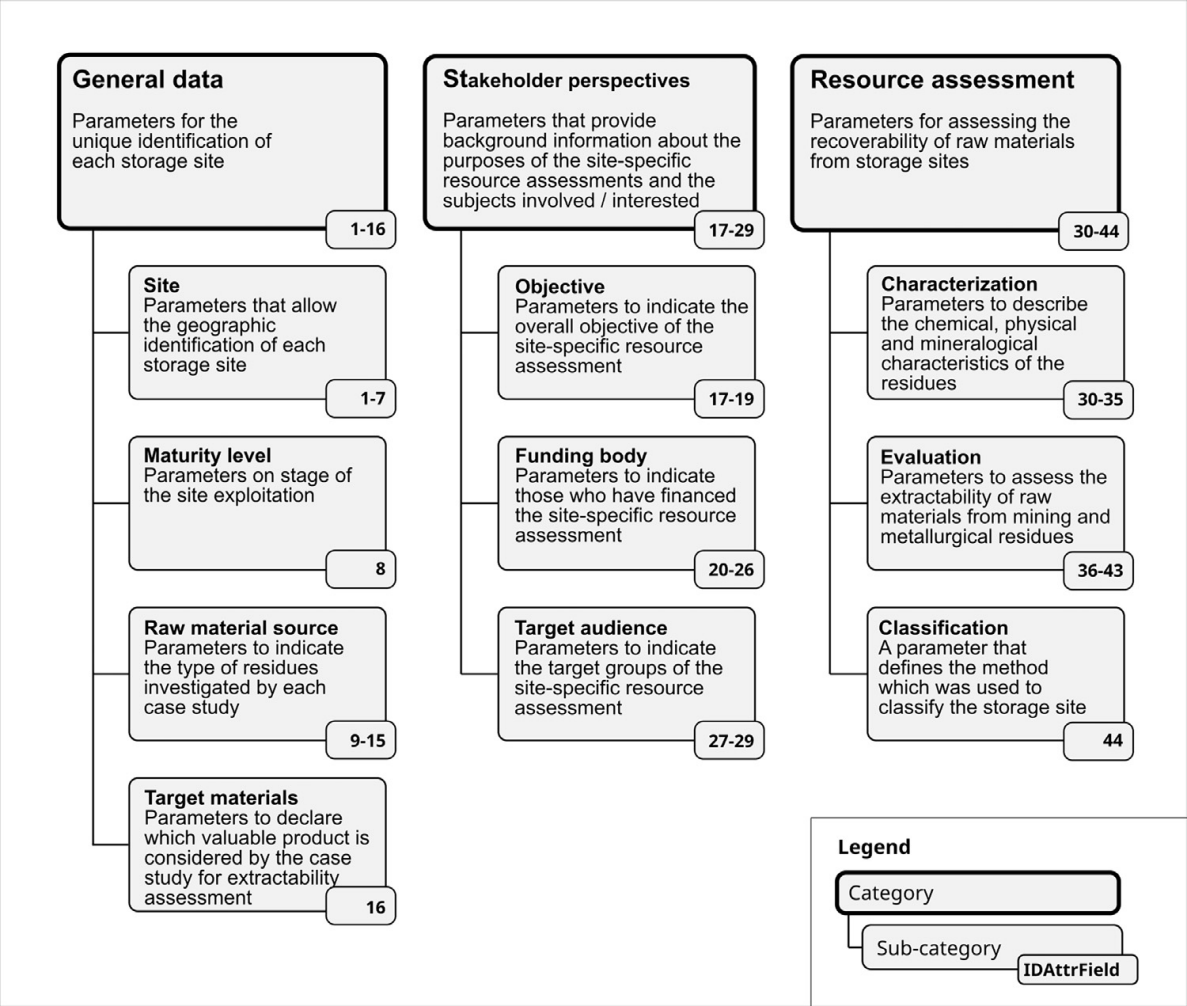
Categories and sub-categories of attribute fields.
• Defined in Table 2. It is noted that the nomenclature of the entries of “AttributeField” is based on the principles of the Infrastructure for Spatial Information in Europe (INSPIRE). For instance, INSPIRE uses the term “includes” if only a Boolean value type can be selected to indicate the consideration of a specific aspect. It is noted that only the data field “ClassificationMethodUsed” is listed in INSPIRE Specifications on Mineral Resources [3], whereas all other data fields are not specified in INSPIRE;

The “sites” layer includes 64 data entries (one per site) with 44 attribute fields. The 44 attribute fields are:

• Thematically clustered into 3 categories and 10 sub-categories (Fig. 3).

**Table 2:** Definition of attribute fields of the „sites“ layer. The table is included in the “site-spec” layer.

IDAttrField	AttributeField	Definition
1	IDSite	A unique identifier for each storage site
2	SiteName	A unique name of the storage site
3	MiningAreaOrRegionName	A unique name of the area or region in which the storage site is located.
4	CountryName	The name of the country in which the storage site is located.
5	ContinentName	The name of the continent in which the storage site is located.
6	WGS84_East	WGS84 longitude East
7	WGS84_North	WGS84 latitude North
8	MaturityLevel	Site exploitation stage
9	includesWasteRocks	A flag indicating if the raw material source is waste rock.
10	includesLowGradeOre	A flag indicating if the raw material source is a low grade ore, which is stored in a stockpile.
11	includesTailings	A flag indicating if the raw material source is a tailing.
12	includesMetallurgicalResidues	A flag indicating if the raw material source is a metallurgical residue.
13	MetallurgicalResidueType	The type of metallurgical residue.
14	includesPreviouslyMinedMinerals	A flag indicating if the assessment includes previously mined materials.
15	includesNonPreviouslyMinedMinerals	A flag indicating if the assessment includes materials that were not mined previously.
16	includesNewMaterials	A flag indicating if the assessment includes new materials.
17	includesMineralRecovery	A flag indicating if the stakeholder objective includes mineral recovery.
18	includesMaterialRecovery	A flag indicating if the stakeholder objective includes material recovery.
19	includesLandRecovery	A flag indicating if the stakeholder objective includes land recovery.
20	includesEnvironmentalRemediation	A flag indicating if the stakeholder objective includes mineral recovery.
21	includesPublicAgency	A flag indicating if the study was financed by a public agency.
22	includesEuropeanUnion	A flag indicating if the study was financed by the European Union.
23	includesNonProfitOrganization	A flag indicating if the study was financed by a non-profit organization.
24	includesMiningCompany	A flag indicating if the study was financed by a mining company.
25	includesPrivateCompany	A flag indicating if the study was financed by a private company.
26	includesUniversityOrResearchUnit	A flag indicating if the study was financed by a university of research center.
27	includesResearch	A flag indicating if the target audience of the resource assessment includes researchers.
28	includesMarket	A flag indicating if the target audience of the resource assessment includes market participants.
29	includesPublicAdministration	A flag indicating if the target audience of the resource assessment includes public administration.
30	includesOreMeasure	A flag indicating if data on the volume or mass of resources and/or reserves are available.
31	includesChemicalSpecification	A flag indicating if the characterization of residues includes chemical specification data.
32	includesParticleSizeAndDistribution	A flag indicating if the characterization of residues includes particle size and distribution data.
33	includesMaterialComposition	A flag indicating if the characterization of residues includes material composition data.
34	includesWaterContent	A flag indicating if the characterization of residues includes water content data.
35	includesLeachates	A flag indicating if the characterization of residues includes leachate data.
36	includesEconomicFeasibility	A flag indicating if the resource recovery evaluation includes economic feasibility.
37	includesEnvironmentalImpact	A flag indicating if the resource recovery evaluation includes environmental impact.
38	includesMarketAcceptance	A flag indicating if the resource recovery evaluation includes market acceptance.
39	includesSocioPoliticalAcceptance	A flag indicating if the resource recovery evaluation includes socio-political acceptance.
40	includesLegalAccessibilityToSource	A flag indicating if the resource recovery evaluation includes legal access to the source.
41	includesTechnicalRecoverability	A flag indicating if the resource recovery evaluation includes technical recoverability
42	includesInfrastructureFeasibility	A flag indicating if the resource recovery evaluation includes infrastructure feasibility.
43	includesLegalCompliance	A flag indicating if the resource recovery evaluation includes legal compliance
44	ClassificationMethodUsed	Name of the code or standard used for resource classification
• Further specified in the “sites_specs” layer, which has 7 attribute fields (see Table 3).

**Table 3:** Definition of the attribute fields of the “sites_spec” layer. This table can be found in the “sites_spec_info” layer.

Field	Definition
IDAttrField	A unique identifier for each attribute field.
Name	The name of the field, as used in the “sites_specs” layer.
Sub-category	Second level categorization of attributes fields, namely the “sub-categories” (see Fig. 3).
Category	First level categorization of attributes fields, namely the “categories” (see Fig. 2).
Definition	A brief definition of the attributes field.
Description	A description of the attribute field.
DataType	Example: “Integer”, “String”, “Boolean”
Codelist	A list of pre-defined values.

#### 3.2.3 Example for a Storage Site in the Database

**Fig. 4:**
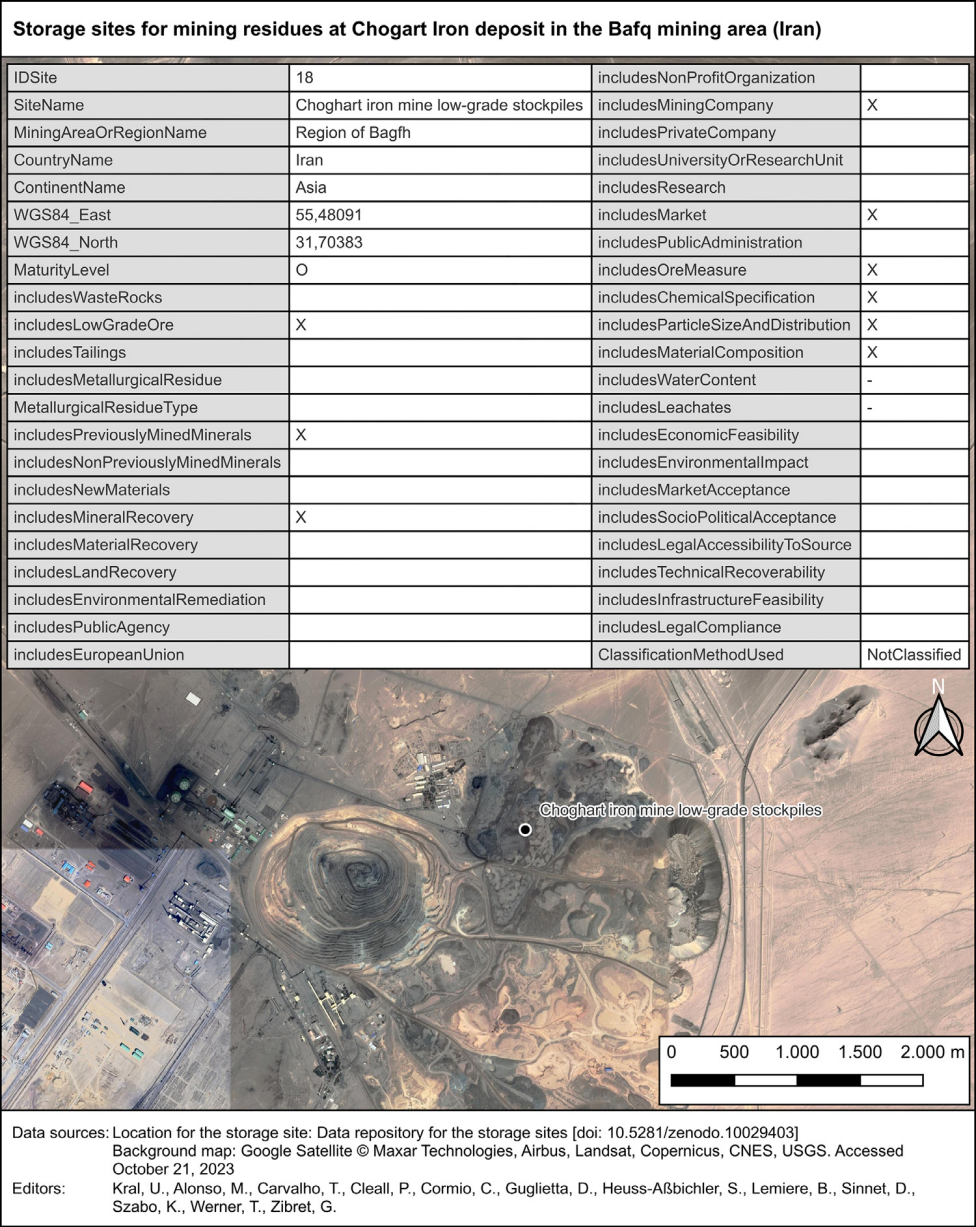
Storage site for mining residues at Choghart iron deposit in the Bafq mining area (Iran) one of 64 storage sites in the “site” layer. Notes: “O” = Operating, “X” = data available, “-“ = not applicable. “Null” = data not available.

The geographical coding of the storage sites is based on point-features. They are located either directly within the spatial extent of the storage site or within the wider mining and production area. The latter was used in those cases where data on the exact location were missing and where the visual detection on satellite images on Earth's surface failed. One example of a storage site, namely the one for mining residues at the Choghart iron deposit in the Bafq mining area (Iran), is presented in Fig. 4.

### 3.3 Geopackage Layer “Bibliography”

The “bibliography” layer includes 63 data entries with 15 attribute fields (Table 4).

**Table 4:** Specification for the attribute table of the “bibliography” layer, which is available in the “bibliography_info” layer.

Field	Definition
IDLiterature	A unique identifier for each bibliographic entry.
ShortReference	First author (and second if no additional co-authors are listed) and year of publication
Authors	List of authors
Year	Year of publication
Title	Title of the document
Journal	Journal name
Publisher	Publisher name
Conference	Conference name
Date	Conference or seminar date
University	University name
ThesisType	Type of academic theses (e.g. Master Thesis)
City	City name
Country	Country name
RetrievedFrom	Uniform Resource Locator (URL)
RetrievedAt	Download date or website access date
DocumentType	Type of the document (Paper/Book, Thesis, Presentation, Conference paper, Journal article, Report, Media release, Website)

## 4 Experimental Design, Materials and Methods

The final geospatial dataset [1] provides a compilation of findings from an evidence-based review on site-specific resource assessment studies. The compilation of findings followed six iterative steps: the definition of parameters, the development and sharing of templates for data collection, the testing of the templates based on 10 different resource assessment studies, the review of 54 additional resource assessment studies, the definition of geographical coordinates for each storage site, and the merging of all the collected data in a single file with an open file format.

These six steps are detailed as follows:

1. At step 1, a spreadsheet was used to draft a list of parameters, which needed to be quantified based on the review of the resource assessment studies (step 3 and 4). First, to group the parameters thematically, three categories and ten sub-categories were defined (Fig. 3). The category “resource assessment” was sub-categorized into “characterization”, “evaluation” and “classification” in alignment with the generic approach for resource assessments, as presented in the Strategic Roadmap on Sustainable Management of Anthropogenic Resources [4]. The parameters for the “resource assessment” category were selected based on a review of the CRIRSCO template [5] and UNFC 2019 [6]. The parameters for the categories “general data” and “stakeholder perspectives” were introduced to place the resource assessments into context. Second, the draft parameter list was continuously updated during the review of the resource assessment studies (step 3). Third, each parameter has been described by a unique identification number, a name, a category and sub-category, a definition, an explanation, a data type and a codelist. The results of step 1 are the layers “site_specs” and “site_specs_info”, included in the dataset [1].
2. At step 2, the “site-specs” layer was used to create a data template spreadsheet, which included a column for each parameter that was identified at step 1. Additionally, a bibliographic template was created to reference the resource assessment studies with key bibliographic data. Finally, the parameter list (step 1) as well as the data and bibliographic templates were saved as online spreadsheets to allow collaborative data compilation among all co-authors.
3. At step 3, the applicability of the data and bibliographic templates (step 2) was tested by manually retrieving relevant information from ten different resource assessment studies. These ten studies refer to the first 10 storage sites in the “sites” layer, corresponding to “IdSite” values 1 to 10. Based on this review approach, the draft parameter list evolved over time (step 1) as well as the data and bibliographic templates (step 2). Multiple repetitions of step 1 to 3 by all co-authors produced a robust parameter list as well as data and bibliographic templates that allowed a systematic, consistent, and comparable compilation of data from resource assessment studies. The final loop resulted in the quantification of the 44 parameters of the first 10 storage sites, which can be found in the “sites” layer [1].
4. At step 4, the co-authors identified additional storage sites, reviewed corresponding resource assessment studies, and manually added the relevant information into the data and bibliographic templates. The selection of storage sites was based on two criteria. The first criterion was the presence and public accessibility of a site-specific resource assessment study. The second criterion was having at least five storage sites per continent in the dataset. The narrative for setting the minimum target was to demonstrate that exploring recovery potential from mining and metallurgical residues is not limited to a single country or continent.
5. At step 5, the storage sites were located in satellite imagery. There were two methods for obtaining the geographical coordinates of each site. If coordinates were already defined in the resource assessment study, these coordinates were manually added to the data template and visually verified with satellite imagery in the Google Earth web application [7]. If coordinates were not given in resource assessment studies, the geographical description (e.g. name of the mine or mining area, name of cities next to the site, maps) was used to identify and verify the site through visual inspection of satellite imagery using the Google Earth web application [7]. In each way, the location of the point-feature on the map is evidence-based, and is placed either directly within the anthropogenic deposit or within the wider mining area.
6. After step 4 and 5 have been completed, the collaborative online spreadsheets (step 3) were converted into comma-separated value (csv) files and added to a single QGIS project file (www.qgis.org). This enabled centralised and structured access to all the data that have been manually compiled from literature review. The Choghart Iron deposit is given as an example for the site-specific data view by the end-user of the dataset (Fig. 4). Finally, the QGIS project file was used to generate a file in geopackage format (https://www.geopackage.org), which is an open file format that can be handled by a wide range of GIS software applications.

Readers are encouraged to share additional reports on site-specific resource assessment studies of anthropogenic deposits with the corresponding author of this paper, further developing the dataset and so contributing to filling the global resource data gap.

## Limitations

The dataset is limited to 64 storage sites for mining and metallurgical residues across all continents. It neither covers all storage sites of a single country nor of the globe.

## Ethics Statement

The authors declare that they have read and followed the ethical requirements for publication in Data in Brief and confirm that the current work does not involve human subjects, animal experiments, or any data collected from social media platforms.

## CRediT authorship contribution statement

**Carlo Cormio:** Conceptualization, Methodology, Data curation, Writing – review & editing, Writing – original draft. **Marta Alonso:** Data curation, Writing – review & editing. **Peter Cleall:** Data curation, Writing – review & editing. **Soraya Heuss-Assbichler:** Conceptualization, Methodology, Data curation, Writing – review & editing. **Daniela Guglietta:** Data curation, Writing – review & editing. **Danielle Sinnett:** Data curation, Writing – review & editing. **Katalin Szabo:** Data curation, Writing – review & editing. **Gorazd Žibret:** Data curation, Writing – review & editing. **Teresa Carvalho:** Conceptualization, Data curation, Methodology, Writing – review & editing. **Ulrich Kral:** Writing – original draft, Data curation. **Tim Werner:** Data curation, Writing – review & editing. **Bruno Lemiere:** Data curation, Writing – review & editing.

## Data Availability

Mining and Metallurgical Residue Database (Reference data) (Zenodo).
